# Air Pollution and Maximum Temperature Are Associated with Neurodevelopmental Regressive Events in Autism Spectrum Disorder

**DOI:** 10.3390/jpm12111809

**Published:** 2022-11-01

**Authors:** Richard E. Frye, Janet Cakir, Patrick J. McCarty, Shannon Rose, Leanna M. Delhey, Raymond F. Palmer, Christine Austin, Paul Curtin, Maayan Yitshak-sade, Manish Arora

**Affiliations:** 1 Rossignol Medical Center, Phoenix, AZ 85050, USA; 2 Department of Applied Ecology, North Carolina State University, Raleigh, NC 27695, USA; 3 Tulane School of Medicine, New Orleans, LA 70112, USA; 4 Arkansas Children’s Research Institute, Department of Pediatrics, University of Arkansas for Medical Sciences, Little Rock, AR 72202, USA; 5 Department of Family and Community Medicine, University of Texas Health Science Center, San Antonio, TX 78229, USA; 6 Department of Environmental Medicine and Public Health, Icahn School of Medicine at Mount Sinai, New York, NY 10029, USA

**Keywords:** autism spectrum disorder, air pollution, neurodevelopmental regression, temperature

## Abstract

Neurodevelopmental regression (NDR) is an enigmatic event associated with autism spectrum disorder (ASD) during which a child loses previously acquired skills and develops ASD symptoms. In some, a trigger which precedes the NDR event, such as a fever, can be identified, but in many cases no trigger is obvious. We hypothesize that air pollution (PM_2.5_) may trigger NDR, especially in those children without an identified trigger. Average daily PM_2.5_, ozone, precipitation and maximum temperature (T_max_) were derived from Environmental Protection Agency models and National Oceanic and Atmospheric Administration monitors based on zip-code information from 83 ASD participants during the six-weeks following the onset month of an NDR event and a reference period defined as one year before and one year after the event. Seasonally adjusted logistic regression (LR) and linear mixed models (LMM) compared cases (with a history of NDR) and matched controls (without a history of NDR). LR models found that the risk of NDR was related to higher PM_2.5_ during 3 to 6 weeks of the NDR event period, particularly in those without a trigger. Overall, both models converged on NDR being related to a higher PM_2.5_ and lower T_max_ both during the NDR event period as well as the reference period, particularly in those without a known trigger. This temporal pattern suggests that environmental triggers, particularly PM_2.5_, could be related to NDR, especially in those without an identifiable trigger. Further studies to determine the underlying biological mechanism of this observation could help better understand NDR and provide opportunities to prevent NDR.

## 1. Introduction

Autism spectrum disorder (ASD) is a behaviorally defined disorder [1] with the most recent Autism and Developmental Disabilities Monitoring Network estimates suggesting 1 in 44 children are affected [1] and a more recent study suggesting that the prevalence might be as high as 1 in 30 [2]. Recent studies suggest that inherited single-gene and chromosomal defects account for a minority of ASD cases [3], and that ASD most likely arises from a complicated interaction between genetic predisposition and environmental exposures [4,5]. Several studies have found links with prenatal air pollution exposure [6], seasonal factors [7], and other environmental exposures [7].

Individuals with ASD demonstrate three developmental trajectories: In the early onset subtype, symptoms are obvious from infancy, perhaps at birth; in the plateau subtype, infants develop normally in the first year of life but then the rate at which they gain skills appears to plateau; lastly in a subset with neurodevelopmental regression (NDR), children develop normally, or near normal, but then lose previously attained skills, usually language and social skills, and develop characteristic symptoms of ASD. 

NDR is often associated factors such as seizure [8] and/or fever [9]. Usually, these factors precede or coincide with the NDR event. For example, NDR is also reported in individuals with mitochondrial disease with an infection preceding by 7–10 days [10] and when ASD develops, with a fever preceding within a similar time period [9]. Thus, these factors are many times thought to be a trigger which may cause or contribute to the NDR event, but the data for this temporal relationship is limited. Many families do not report any obvious associated factors or ‘triggers’ when a NDR event occurs.

A meta-analysis has shown that NDR is more common in children with ASD that were also diagnosed with mitochondrial disease [11]. Mitochondrial dysfunction is seen in multiple disorders closely linked to ASD [12,13,14] and animal models of ASD [15,16]. Our previous studies of children with ASD [17,18] and in vitro cell ASD models [19,20,21,22,23,24] suggest mitochondria may be sensitive to environmental exposures such that environmental agents may deplete the ability of the mitochondria to produce adenosine triphosphate (ATP), the energy carrier of the cell. Depletion in ATP production can result in cellular dysfunction and even cell death (apoptosis).

We have demonstrated that exposure to air pollution, as measured from Environmental Protection Agency’s (EPA) local monitors, is associated with long-term variations in mitochondrial physiology in children with ASD [17]. Particulate matter (PM) contains microscopic solids or liquid droplets that are so small that they can be inhaled and cause health problems. PM_2.5_ describes fine inhalable particles, with diameters that are generally 2.5 μm and smaller. PM has been associated with a wide range of health effects in children, including prenatal exposure being linked to adverse effects on children’s respiratory, immune, nervous, and cardiovascular system health [25]. Post-natal exposure to PM has been linked to detrimental neurodevelopmental [26] and respiratory outcomes [27], although the association appears to be weaker as compared to prenatal exposures.

Thus, we suspect that PM_2.5_ could trigger NDR, potentially through disruption of mitochondrial function. Furthermore, it is possible that other environmental factors such as ozone, precipitation and temperature fluctuations could also affect the mitochondria, thereby triggering NDR in children with ASD, particularly in those without an identifiable trigger. Thus, we hypothesize that exposure to PM_2.5_, ozone, precipitation and/or temperature fluctuations could trigger NDR in children with ASD that do not have an identifiable trigger.

## 2. Materials and Methods

### 2.1. Participants

The participants were recruited for a natural history study in ASD registered in clinicaltrials.gov (NCT02000284). Methods can be found in previous publications using this participant cohort [17,18], although they are briefly outlined here. The protocol was approved by the Institutional Review Board at the University of Arkansas for Medical Sciences (Little Rock, AR). Parents of participants provided written informed consent.

Exclusion criteria were (i) chronic treatment with medications that would detrimentally affect mitochondrial function such as antipsychotic medications; (ii) vitamin or mineral supplementation exceeding the recommended daily allowance, and (iii) prematurity. Premature children were excluded as they can have multiple comorbid medical and developmental conditions that are specifically related to their prematurity rather than to physiological processes typically associations with ASD.

Inclusion criteria included the ability to tolerate phlebotomy and a diagnosis of ASD. The ASD diagnosis was documented by one of the following: (i) a gold-standard diagnostic instrument such as the Autism Diagnostic Observation Schedule and/or Autism Diagnostic Interview-Revised (ADI-R); (ii) the state of Arkansas diagnostic standard, defined as agreement of a physician, psychologist and speech therapist who specializes in ASD; and/or (iii) Diagnostic Statistical Manual of Mental Disorders, Fourth Edition, Text Revision (DSM) diagnosis by a physician along with standardized validated questionnaires which have good correspondence to the gold-standard instruments including the Social Responsiveness Scale (SRS) [28,29], the Social Communication Questionnaire [30,31,32] and the Autism Symptoms Questionnaire [33] and diagnosis confirmation by the Principal Investigator (first author) who specializes in the diagnosis and treatment of children with ASD. In our recent clinical trial [34], methods (ii) and (iii) were validated by re-evaluating a portion of the participants diagnosed with methods (ii) and (iii) using the ADI-R and finding that their ADI-R scores fell well within the diagnostic criteria for ASD.

The NDR history was obtained using the Developmental and Neurobehavioral Regression (DANR) questionnaire which has been developed as part of our ASD research program. The DANR records detailed information about NDR including specific questions on premorbid functioning before the regression, duration of the regression, specific skills lost and when the skills were regained, whether there was a single or multiple regressions, and any known trigger such as illness, fever, or seizure.

### 2.2. Exposure Variables

Parents provided the zip code for the participant’s residence from birth to current age on a standard medical history intake form. If an individual moved during the observation period, the measurements from the location during the time they lived at the location were used. Six individuals moved once during the observation period. Four of the individuals were in the NDR without trigger group and moved 5, 6, 7 and 7 months before the NDR event. Two of the individuals were in the NDR with trigger group and moved 3 and 5 months before the NDR event.

Regional daily PM_2.5_ measures were obtained from the EPA’s Air Quality System (AQS) like other studies investigating ASD and PM_2.5_ exposure [35]. The AQS includes ambient air pollution and meteorological measurements collected by EPA, state, local, and tribal air pollution control agencies from over thousands of validated outdoor monitors which meet the EPA’s monitoring network requirements [36]. The monitors provide information on daily mean concentrations of the pollutants.

**Figure 1:**
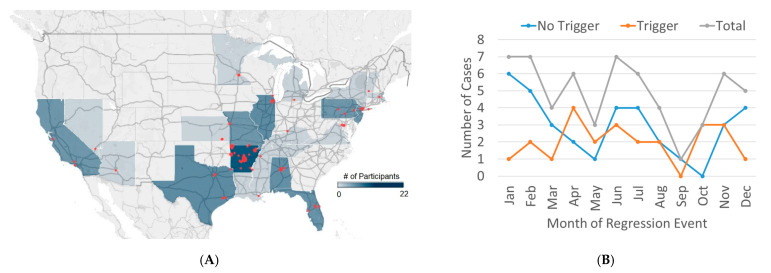
(**A**) Distribution of participants in this study. States are shaded by number of unique ZIP codes, and red dots are sized by number of patients within each zip code. (**B**) Seasonal timing of neurodevelopmental regression events for those with a potential trigger and those without an identifiable trigger.

Although some previous studies have modeled air pollution exposure using geospatial modeling such as land use regression, such models are best utilized when the population studied is in a relatively circumscribed area where monitors are sparse. In this study, the participants lived across the entire United States, mostly in urban areas where air monitors are relatively dense and the participants themselves are relatively widely separated (Figure 1A).

For the month of regression and the twelve months before and after, downscaled daily models of US FIPS code scale average PM_2.5_ and 8-h daily maximum Ozone were downloaded from the EPA’s Remote Sensing Information Gateway [37,38]. The FIPS code level data are a product of a Bayesian space-time downscaled predicted surface for air quality fusing together monitored data from the National Air Monitoring Stations/State and Local Air Monitoring Stations (NAMS/SLAMS) with 12 km gridded output from the Models-3/Community Multiscale Air Quality (CMAQ) model. The daily FIPS code level fused data for each pollutant were then averaged across the zip code that each participant resided in when the first regression took place. Daily precipitation and maximum temperature (T_max_) for the nearest weather station were downloaded from the National Oceanic and Atmospheric Administration [39], then merged with the zip code level daily PM_2.5_ and Ozone data for 27 months centered on the regression month.

To determine if participants from the different groups were located in substantially different areas of urbanization, rural-urban commuting area (RUCA) codes were assigned based on zip-code. If the participant lived in two different areas during the exposure period, the codes were averaged. RUCA codes classify US census tracts using measures of population density, urbanization, and daily commuting and are whole numbers ranging from 1 to 10 based on the size and direction of the largest commuting flow in the area.

### 2.3. Data Analysis

To determine the relationship between NDR and environmental variables, we used a case–control design that compared cases of individuals with ASD who experienced NDR to control cases of children with ASD without reported NDR. The NDR event was defined using the DANR questionnaire described above. All questionnaire data was reviewed by the examiner who collected the data (L.M.D.) and the first author (R.E.F.) and follow-up questions were performed if information was unclear or ambiguous. NDR was defined as a categorical variable. The start of the NDR event was defined by the month in age when the event occurred. Since the specific day in which the event started could have occurred at any time during the NDR event period month, a 6-week window defined the NDR event in order to capture changes that could have occurred at the end of the reported month. This six-week period was divided into three two-week blocks using three dummy variables. The reference period was defined as the exposure one year prior and one year after the NDR event period.

The primary analysis was a case–control design. Individuals without a history of NDR were matched to those with NDR based on age of diagnosis. Age of diagnosis was selected to control for any variation in diagnosis due to different developmental profile (NDR vs. not NDR). For the participants without a NDR event, environmental variables were derived from the same time period in life as the match participants with NDR. As there were fewer participants without NDR, we used a matching with replacement approach. Specifically, some participants without NDR had to be matched to several participants with NDR. Sex and race were entered into models initially but did not confound the associations for any of the models and were therefore removed.

A multivariable logistic regression (LR) model was used to understand which environmental variables in combination may be related to developing ASD through an NDR profile. Outcome was a dichotomous NDR vs. no NDR. Predictor variables included season, the NDR event period (divided into 2-week blocks), the four environmental variables and the interaction between the environmental variables and the NDR event period (i.e., the time period in which the NDR event occurred). LR models were simplified to remove non-significant non-dependent variables and odds ratios (ORs) were calculated.

To assess the mean difference in PM_2.5_, ozone, precipitation and T_max_ (exposure variables) related to NDR, a single-pollutant analysis of variance (ANOVA) was implemented using a mixed-model approach with adjustment for season, and a random effect of subject to control for repeated effects of subject level mean and variance with an autoregressive moving average covariance structure. The ANOVA determined whether changes in environmental exposures during the NDR event, relative to the reference period, were different between the cases and controls. This difference between cases and controls was tested by adding an interaction between NDR exposure period and NDR status (case vs. control). If the interaction was significant, then the cases and controls were analyzed separately to determine whether exposures during the NDR event period were different than the reference period. If the NDR event period was significant, each of the two-week blocks was compared to the reference period by planned post hoc contrasts using the least significant difference method. In addition, a linear and curvilinear trend was examined during the NDR period.

As previously mentioned, those with NDR may have a known trigger (e.g., seizure, fever) associated with the event or a trigger may not be obvious. We determined whether those with and without a trigger have different environmental pollutant profiles during the NDR event period. The trigger was modeled as a dichotomous variable (trigger, no trigger). Both LR and ANOVA models were used. For the LR analyzed those with and without a trigger were analyzed in single-pollutant LR models. Next, for the ANOVA, the interaction of the trigger with the NDR event period was tested to determine if the environmental variable during the six-week NDR event period, in comparison to the reference period, was different in those with and without a trigger. No controls were used in this analysis, rather the two subgroups (trigger vs. no trigger) of NDR participants were compared. If the interaction was significant, the group of participants with and without a trigger were analyzed separately to determine the difference in the environmental exposure during the NDR event period relative to the reference period for each group separately. If the NDR event period was significant, each of the two-week blocks was compared to the reference period using planned post hoc contrasts using the least significant difference method.

## 3. Results

### 3.1. Participant Characteristics

Table 1 provides demographics of the participants. Fifty-eight participants were identified with NDR, with 25 (43%) having an associated trigger. Twenty-five participants were identified that did not have NDR. The cohort was mostly male and white although those with no history of NDR had slightly more females. There were 4 children of Asian and 4 children of middle eastern race. There was one Hispanic child. There was no difference in Rural-urban commuting area codes (RUCA) between groups, suggesting similar urban environments. Very few participants lived in rural settings. Age at regression was borderline significantly older in the children with ASD without a trigger as compared to those with a trigger [t(57) = 1.93, *p* = 0.06]. Of those with a trigger, most regressed with fever (56%) and/or illness (52%). Illness, when details were given, was always an infectious type of illness. Two patients (8%) experienced a seizure with onset of the NDR.

The distribution of the participants is depicted in Figure 1A. Although most were near the study location in Little Rock AR, patients represented many of the other larger state of the Union. Examining the seasonal timing of the NDR event (Figure 1B) demonstrates that few events occurred in late summer/early fall. Those without an identifiable trigger appeared to have two peaks, one in the winter (Jan) and one in the summer (June, July) whereas those with an identifiable trigger did not seem to have a clear peak.

### 3.2. Case-Control Comparisons

#### 3.2.1. Multivariate Logistic Regression Models

A LR of NDR vs. no NDR cases was conducted including the four environmental variables (See Table 2). Season was significantly related to NDR risk [F(3,68056) = 67.50, *p* < 0.001] with winter demonstrating a decreased risk [OR 0.873, 95% CI (0.826, 0.925)] and spring and summer demonstrating an increased risk [Spring: OR 1.233, 95% CI (1.174, 1.296); Summer: OR 1.222, 95% CI (1.162, 1.285)] relative to the fall season. For the overall exposure period measured (one year prior to and one year after the NDR event as well as the NDR event), PM_2.5_ was associated with an increased odds of NDR [OR 1.025, 95% CI (1.022; 1.029) per 1 µg/m^3^], and lower odds of NDR was associated with precipitation [OR 0.887, 95% CI (0.850, 0.925) per 1 mm], Ozone [OR 0.984, 95% CI (0.982, 0.985) per ppb], and T_max_ [OR 0.991, 95% CI (0.984, 0.998), per 1 °F]. Testing whether changes during the NDR event period were related to the risk of being an NDR case found that both PM_2.5_ and T_max_ were related to exposures specifically during the NDR event period. Specifically, the risk of NDR was increased when PM_2.5_ was higher during weeks 3–6 of the NDR event period [Weeks 3–4 OR 1.025 (1.000, 1.050); Weeks 5–6 OR 1.043 (1.017, 1.070)] and when T_max_ was lower during weeks 5–6 of the NDR event period [OR 0.991 (0.984, 0.998)].

#### 3.2.2. Analysis of Variance Models

To examine the difference in mean exposure between the case and control groups, daily exposure data (PM_2.5_, ozone, precipitation, T_max_) for children with ASD was compared between those with and without a history of NDR. Essentially, any change in the environment during the NDR event period in the NDR cases was compared to changes at a similar age in the non-NDR controls.

**Table 2:** Logistic Regression Model for All Neurodevelopmental Regression Participants.

Model Term	β	Std. Error	t-Value	*p*	Odds Ratio (95% CI)
Intercept	0.782	0.2494	3.134	0.002	2.185 (1.340, 3.563)
**Season (Fall Reference)**					
Winter	−0.134	0.0287	−4.677	<0.001	0.874 (0.826, 0.925)
Spring	0.210	0.0253	8.292	<0.001	1.233 (1.174, 1.296)
Summer	0.201	0.0255	7.852	<0.001	1.222 (1.162, 1.285)
**NDR Event (Compare to Reference Time Period)**					
Weeks 1 to 2	0.046	0.2486	0.184	0.854	1.047 (0.643, 1.704)
Weeks 3 to 4	0.056	0.2508	0.224	0.823	1.058 (0.647, 1.729)
Weeks 5 to 6	0.177	0.2793	0.633	0.527	1.193 (0.690, 2.063)
**Air Pollution (PM2.5)**					
Overall Exposure	0.025	0.0016	15.815	0.000	1.025 (1.022, 1.029)
NDR Weeks 1 to 2	0.019	0.0137	1.413	0.158	1.019 (0.993, 1.047)
NDR Weeks 3 to 4	0.024	0.0125	1.955	0.051	1.025 (1.000, 1.050)
NDR Weeks 5 to 6	0.042	0.0130	3.223	0.001	1.043 (1.017, 1.070)
**Ozone**					
Overall Exposure	−0.017	0.0007	−22.937	0.000	0.984 (0.982, 0.985)
**Precipitation**					
Overall Exposure	−0.120	0.0216	−5.553	<0.001	0.887 (0.850, 0.925)
**Maximum Temperature**					
Overall Exposure	0.001	0.0007	1.164	0.244	1.001 (0.999, 1.002)
NDR Weeks 1 to 2	−0.003	0.0031	−1.048	0.295	0.997 (0.991, 1.003)
NDR Weeks 3 to 4	−0.005	0.0033	−1.423	0.155	0.995 (0.989, 1.002)
NDR Weeks 5 to 6	−0.009	0.0036	−2.509	0.012	0.991 (0.984, 0.998)

PM_2.5_ was significantly influenced by season [F(1,62895) = 2,162.04, *p* < 0.001] and was significantly different between NDR and non-NDR [F(1,62895) = 48.08, *p* < 0.001]. Those that experienced NDR demonstrated a higher average exposure to PM_2.5_ (both during the NDR event period and reference periods) [Mean (SE): NDR 12.2 (0.04) vs. No NDR 11.2 (0.04)]. PM_2.5_ during the NDR event period, as compared to the reference period, was different for those that experienced an NDR event period as compared to those that did not have a reported NDR event period [F(3,6095) = 3.28, *p* = 0.02]. 

**Figure 2:**
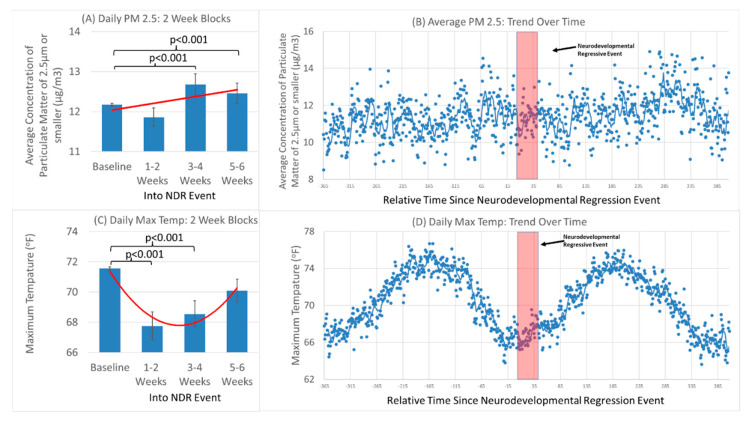
Average concentration of particular matter in the air of 2.5 µm or smaller and maximal daily temperature during a neurodevelopmental regressive event in children with autism spectrum disorder. (**A**,**C**) Mean and standard error of two-week blocks of daily measurements across all ASD participants with a history of NDR. Red lines provide linear and curvilinear line fits to the daily data. (**B**,**D**) Average daily measurements across all ASD participants with a history of NDR. Red box represents the NDR event period.

To understand this interaction better, the case (NDR) and control (No NDR) groups were analyzed separately. Those that experienced an NDR event demonstrated a significant effect of season [F(4,44772) = 1949,3, *p* < 0.001] and a significant change in PM_2.5_ during the NDR event period relative to the reference period [F(3,44772) = 6.47, *p* < 0.001]. As seen in Figure 2A,B, NDR was associated with a PM_2.5_ peak in the weeks 3 to 6 of the NDR event period [Weeks 3–4 Mean Difference 0.64 (0.24) F(1,44772) = 7.11, *p* = 0.007; Week 5–6 Mean Difference 0.72 (0.24) F(1,44772) = 9.00, *p* = 0.003]. Further analysis demonstrated that during the NDR event period, there was a linear progressive increase in PM_2.5_ [Increase by 0.015 (SE 0.005) ug/m^3^/day F(1,44726) = 9.58, *p* < 0.01] (Figure 2A red line). Those without a history of NDR demonstrated relatively stable PM_2.5_ that did not differ significantly in the matched NDR event period as compared to the reference period. The PM_2.5_ analysis was not altered when ozone, precipitation or T_max_ were included as a covariates in the model.

T_max_ was different between the children that experienced NDR as compared to those that did not. There was an effect of season [F(4,64017) = 14,410.8, *p* < 0.001] and NDR [F(1,64017) = 46.53, *p* < 0.001]. Those that experienced NDR demonstrated an average lower T_max_ [Mean (SE): NDR 69.5 (0.01) vs. No NDR 70.5 (0.12)]. The change in T_max_ during the NDR event period was different for those with a history of NDR as compared to those without a history of NDR [F(3,64017) = 7.50, *p* < 0.001].

Analysis of only the individuals experiencing an NDR event demonstrated a significant effect of NDR event period [F(3,46866) = 21.95, *p* < 0.001] and season [F(4,46866) = 11,254.3, *p* < 0.001]. As seen in Figure 2C,D, there was a significant drop in T_max_ in the first 4 weeks of the NDR event period [Weeks 1–2 Mean Difference 3.22 (0.50) F(1,46866) = 41.47, *p* < 0.001; Week 3–4 Mean Difference 2.53 (0.50) F(1,46866) = 25.60, *p* < 0.001]. This followed a curvilinear relationship [Linear Trend F(1,46822) = 72.59, *p* < 0.001; Curvilinear Component F(1,46822, 56.52, *p* < 0.001] (Figure 2C red curve). Those that did not have a history of NDR demonstrated relatively stable T_max_ during the matched NDR event period that did not significantly differ from the reference period.

Neither ozone nor precipitation appears to be different between those with and without NDR.

### 3.3. Difference in Environmental Variables Related to Potential Trigger

Some of the families reported a potential triggering event related to the NDR event. Because air pollution is an environmental toxin, can affect the mitochondrial, and is easily overlooked, we hypothesized that PM_2.5_ was the trigger for the subset of participants with NDR that did not have an identified trigger.

#### 3.3.1. Multivariate Logistic Regression Models

ASD participants with NDR without a trigger were examined (See Table 3). Season was significant related to NDR risk [F(3,51132) = 77.50, *p* < 0.001] with winter demonstrating a decreased risk [OR 0.728, 95% CI, (0.682, 0.778)] and spring and summer demonstrating an increased risk [Spring: OR 1.094, 95% CI (1.033, 1.159); Summer: OR 1.246, 95% CI (1.177, 1.319)] relative to the fall season. The odds of having NDR without a trigger was significantly elevated when PM_2.5_ was higher [OR 1.041, 95% CI (1.037, 1.044), per per 1 µg/m^3^] and T_max_ lower [OR 0.992, 95% CI (0.990, 0.993), per 1 °F] during the overall exposure period (both reference period and NDR event). During the NDR event period, the risk of being an NDR case without a trigger was related to PM_2.5_, T_max_ and Ozone. The risk of being an NDR case with a trigger was higher when PM_2.5_ was elevated during the entire NDR event period [Weeks 1–2 OR 1.046 (1.013, 1.080); Weeks 3–4 OR 1.034 (1.004, 1.064); Weeks 5–6 OR 1.061 (1.027, 1.096)], when T_max_ was lower during the entire NDR event period [Weeks 1–2 OR 0.984 (0.975, 0.992); Weeks 3–4 OR 0.984 (0.975, 0.993); Weeks 5–6 OR 0.969 (0.958, 0.979)] and when ozone was elevated during Weeks 3–4 of the NDR event period [Weeks 3–4 OR 1.009 (0.997, 1.022)].

For ASD participants with NDR with a known trigger (See Table 4), season was significantly related to NDR risk [F(3,43080) = 41.47, *p* < 0.001], with winter demonstrating a decreased risk [OR 0.883 (0.819, 0.952)] and spring and summer demonstrating an increased risk [Spring: OR 1.265 (1.184, 1.351); Summer: OR 1.192 (1.116, 1.274)] relative to fall season. For the overall exposure period (both during the NDR event and reference periods), the risk of having NDR with a trigger was significantly elevated when T_max_ was elevated [OR 1.012 (1.010, 1.014)], when Ozone was lower [OR 0.971 (0.969, 0.973)] and when precipitation was lower [0.729 (0.687, 0.775)]. The risk of being an NDR case for those with a known trigger was not associated with changes in environment during the NDR event.

#### 3.3.2. Analysis of Variance Models

We compared whether PM_2.5_ during the NDR event was different in those with ASD who did not have a trigger as compared to those who had a triggering event. Season showed a significant effect [F(1,44768) = 19399.7, *p* < 0.001]. There was a significant difference in average PM_2.5_ in those that experienced a trigger as compared to those that did not [F(1,44768) = 51.78, *p* < 0.001] with those with a trigger demonstrating a higher overall mean PM_2.5_ [Mean (SE): Trigger 12.78 (0.06), No Trigger 11.27 (0.05)]. PM_2.5_ during the NDR event, relative to the reference period was different between those that had a trigger as compared to those that did not have an identified trigger [F(3,44768) = 3.11, *p* < 0.05].

**Figure 3:**
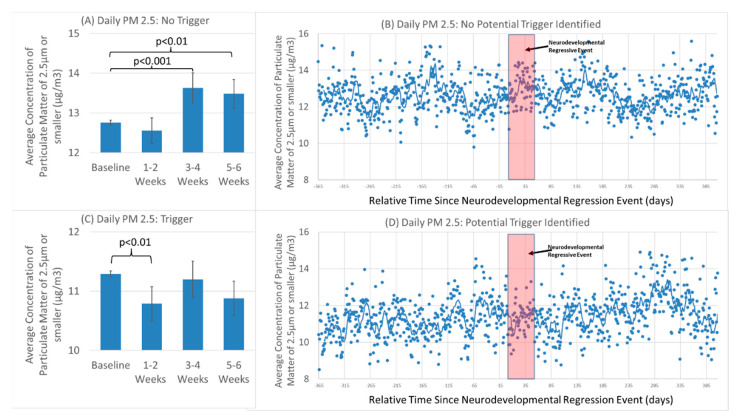
Average concentration of particular matter in the air of 2.5 µm or smaller during the neurodevelopmental regressive event in children with autism spectrum disorder (**A**,**B**) without and (**C**,**D**) with an identified potential trigger. (**A**,**C**) Mean and standard error of two-week blocks of daily measurements. (**B**,**D**) Average daily measurements. Red box represents the NDR event.

Separate analyses were conducted for those participants with and without an identified trigger. Analysis of individuals without a reported trigger demonstrated a significant effect of season [F(4,26616) = 1,627.3, *p* < 0.001] and the NDR event [F(3,26616) = 6.38, *p* < 0.001]. PM_2.5_ was elevated in the weeks 3 to 6 of the onset of the NDR event [Weeks 3–4 Mean Difference 0.91 (0.33) F(1,26616) = 7.60, *p* = 0.005; Week 5–6 Mean Difference 1.10 (0.33) F(1,26616) = 11.11, *p* < 0.001] (Figure 3A,B), similar to the overall findings the NDR group.

Analysis of the individuals with a reported trigger demonstrated a significant effect of season [F(3,18149) = 164.5, *p* < 0.001] and NDR event [F(3,18149) = 2.87, *p* < 0.05]. PM_2.5_ demonstrated a drop in the first 2 weeks of the NDR event [Mean Difference 0.95 (0.34) F(1,18149) = 7.81, *p* = 0.005] (Figure 3C,D). The effect of trigger on PM2.5 did not change when ozone, precipitation or maximum temperature are included as a covariates.

This effect of trigger was not significant for the T_max_ model.

## 4. Discussion

This study hypothesized that common environmental exposures, such as air pollution, could trigger NDR in children with ASD, especially those without an identifiable trigger. The 6 weeks from the start of the reported month of age in which the NDR event occurred was examined to determine if changes in air pollution (PM_2.5_), ozone, precipitation and maximum temperature, all factors that have been linked to human health, were altered during the NDR event period. As a control comparison we matched individuals with ASD who did not experience a NDR event to ensure that changes in environmental variables found were not related to ASD in general but rather specific to the NDR event. Two environmental variables were consistently found to be associated with the NDR event, particularly in those without an identifiable trigger, a spike in PM_2.5_ and a depression in T_max_. The association with PM_2.5_ adds to the emerging literature that consistently finds this as an important risk factor for ASD [6] while the relation to temperature is a rather novel finding. Additionally, the relationship between NDR and environmental factors is a new finding that has significant implications for prevention and understanding the etiological factors associated with ASD.

### 4.1. Air Pollution Effects on Children with Autism Spectrum Disorder

Several recent studies have demonstrated the association between air pollution and ASD [40] with some studies demonstrating factors which mitigate this association, including copy number variation [41], maternal folate intake [42] and MET receptor tyrosine kinase polymorphism [43]. While many studies find the association between air pollution and ASD to be strongest during the prenatal period [44], other studies point to the post-natal period, particularly the first 9 months [45] and first year [46] of life and associated with nitric oxide and dioxide but not PM [47]. However, air pollution studies have failed to examine the post-natal developmental trajectory or account for individuals with NDR. The data we present here provides novel insight to the potential specific effects of air pollution as it relates to ASD and suggests that future studies should account for the post-natal variation in the development of ASD symptoms and perhaps separate those children who experienced NDR from those that do not have such a history.

### 4.2. The Potential Contribution of Temperature

The NDR event was found to be associated with a drop in T_max_. This can be explained by changes in human behavior due to cooling in both the winter and the summer, the times of peak NDR without a trigger cases. In the winter, when the temperature drops, more fuel that has the potential to pollute the air is used to heat. Cars stand idle to defrost them before driving and wood, oil and natural gas are used more to heat. Thus, a cold snap in the winter can result in increased air pollution. The notion of increased use of fuels resulting in increased air pollution is consistent with the NDR occurring, on average, during the baseline temperature trough (Figure 2D).

In the summer, when the temperature drops, children are taken outside to play to enjoy the weather as a reprieve from the heat of summer. Commonly physical activity is increased when young children run as part of outdoor play. The increased physical activity could increase respiratory rates and thus increase the intake of polluted air. Under a changing climate, the probability of high temperature events (logically relieved by a few days of lower temperatures), are projected to increase [48]. This phenomenon of summer temperature variability and expanding warm seasons in combination with higher air pollution concentrations associated with urban areas could result in more days in each year that would meet these conditions. The outdoor recreational behavior of small children and their stage of development and potential for asymptomatic underlying mitochondrial dysfunction due to prenatal pollution exposure [17] make them a uniquely sensitive and socially vulnerable population at risk to the adverse effects of air pollution.

### 4.3. Potential Biological Mechanisms of Neurodevelopmental Regression

The biological mechanism for NDR is not known but it is known that individuals with mitochondrial dysfunction are susceptible to environmental triggers, often resulting in NDR. Shoffner et al. [9] found that the majority of children with ASD and mitochondrial disease developed ASD symptoms after a sudden rapid NDR which was sometimes associated with a fever that usually preceded the NDR event [9]. Individuals with mitochondrial disease are known to experience NDR with illness [10] and a meta-analysis found that NDR was more common in children with ASD and mitochondrial disease than in ASD children without mitochondrial disease [11]. A recent study suggests that those with ASD and NDR may have unique abnormalities in mitochondrial physiology as compared to those with ASD without NDR [49]. Thus, NDR may be a hallmark of abnormal mitochondrial physiology in ASD.

### 4.4. Unique Changes in Mitochondrial Dysfunction Are Linked to Neurodevelopmental Regression in Autism Spectrum Disorder

This notion that physiological stressors can compromise mitochondrial function is consistent with our in vitro lymphoblastoid cell line (LCL) model of ASD where a subset of LCLs with mitochondrial dysfunction are found to be very sensitive to increases in physiological stress [19,21,22,23,50,51,52]. Indeed, we have demonstrated that this subset of LCLs that are sensitive to in vitro physiological stress are also sensitive to environmental agents associated with ASD, including trichloroacetaldehyde hydrate [20] and ethylmercury [52], and enteric short-chain fatty acids propionate [34] and butyrate [50]. Furthermore, we have demonstrated that prenatal exposure to PM_2.5_ results in long-term changes in mitochondrial function consistent with the pattern of mitochondrial dysfunction associated with NDR [17].

### 4.5. ‘Triggers’ Associated with Neurodevelopmental Regression

The phenomenon of NDR is one that is evolving. Analysis of studies of NDR in ASD demonstrates that it appears to be on a continuum with some having sudden regression while other demonstrating a more protracted course in the loss of skills [53]. Others have pointed to the fact that stagnation or a plateau in development can be confused with loss of skills and that many children with neurodevelopmental disorders such as ASD have loss of skills well after the diagnosis, making regression possibly the rule rather than the exception in many neurodevelopmental disorders [54]. However, the sudden loss of skills is pathognomonic for an underlying severe pathophysiological process linked to mitochondrial disease preceded (i.e., triggered) by an inflammatory process [9,10] or an autoimmune encephalitis [55]. In children with ASD, a fever preceding the NDR event appears to be linked to underlying mitochondrial disease [9].

This study was not able to provide a temporal link between air pollution and the NDR event, but rather found that the peak in air pollution occurred coincident with the NDR event. This could very well be due to the retrospective report and the limited accuracy of timing given the one-month resolution of the parental report. As mentioned in the previous section, studies have shown that a subset of children with ASD have mitochondria that demonstrate elevated respiratory rates which make them sensitive to physiological stress [19,21,22,23,50,51,52]. Laboratory studies have demonstrated that this unique signature of mitochondrial dysfunction can be induced by exposure to low levels of oxidative stress [24] and studies in children with ASD demonstrate that this pattern of mitochondrial dysfunction is related to prenatal exposure to air pollution [17] as well as nutritional metals [18]. Thus, it is very possible that air pollution contributed to mitochondrial dysfunction and loss of ATP production in those children that have vulnerable mitochondria.

It is hypothesized that mitochondria are predisposed to be sensitive to stressors post-natally because of prenatal exposures. However, whether this is one exposure or a series of exposures, it is not clear. There is growing evidence that many exposures which can affect the mitochondria are linked to ASD [17], including common drugs such as acetaminophen [54]. Post-natal exposures to many other common toxins have been linked to ASD [56] suggesting that the effect of environmental toxins on the mitochondria could be cumulative over time, with one particular exposure pushing the physiology of the mitochondrial pasts its ability to compensate and resulting in the inability of the mitochondrial to produce the energy needed to support central nervous system function. This would be consistent with the time course of PM_2.5_ in Figure 2B and Figure 3B as there does appear to be other PM_2.5_ peaks before and after the NDR event and our analysis that demonstrated that elevated PM_2.5_ over the entire observation period increased the risk of developing ASD. Thus, in this sense the PM_2.5_ peak may be a contributor to other chronic exposures as well as other developmental factors such as the optimal vulnerability of brain development. Further research will indeed be needed to answer these questions.

Lastly, it has been mentioned that fever may trigger a NDR even in children with mitochondrial disease [9,10]. However, one of the curious phenomena in ASD research is that some individuals with ASD have improvements in behavior with fever [57]. This would seem counter to the notion that fever may induce NDR. However, a large study of those with ASD as well as typically developing children demonstrated that only 2% of children with ASD demonstrated this positive response and that, most commonly, fever resulting in a more negative response in children with ASD as compared to typically developing children [58]. Additionally, fever can be produced by mitochondrial uncoupling, a process that can reduce oxidative stress at the inner mitochondrial membrane at the expense of but decreasing efficiency of ATP production [59]. As a subset of individuals with ASD already appear to have uncoupled mitochondria, it would make sense that further uncoupling of the mitochondrial would have variable effects on individuals with ASD as a group [60].

### 4.6. Limitations

This study is the first to examine NDR in the context of air pollution but has several limitations. The study participants were derived from a natural history study of individuals with ASD. As such, much of the data has been obtained retrospectively, including the information on NDR. Thus, a prospective confirmatory cohort in the future would be preferable. Additionally, typically developing controls were not examined in this study. Including typically developing controls, particularly siblings, could provide a strong detailed analysis of metabolic and genetic markers that might help explain the physiological underpinnings of NDR and provide biomarkers for the identification of children at risk for NDR. The sample size of this study is limited, so the effects of certain factors such as sex may not be detected given the limited number of females in the study.

## 5. Conclusions

One of the critical knowledge gaps in understanding the relationship between environmental exposures and the etiology of ASD is understanding the timing of the effect of environmental exposures and the subset of children with ASD who may be affected by these environmental factors. This study suggests that there is a subset of children with ASD who might be sensitive to common environmental factors. As these children usually have near normal development prior to the NDR event, identifying these children early, before the event occurs, could allow mitigation treatments to be utilized to prevent the development of ASD in this subset of children.

Future research will be needed to replicate these findings and examine the effects of other time periods rather than specifically the NDR period. Indeed, it appears that those children who experienced the NDR event did have higher exposure to PM_2.5_ during the year before and after the NDR event, suggesting that their physiological systems may have been under greater chronic stress in general while the event occurred. As prenatal factors also can have long-lasting effects on physiology in children with ASD, such factors may predispose children to be sensitive to an infectious or common environmental trigger [17,18]. Further studies with larger cohorts will be needed to address these questions.

## Figures and Tables

**Table 1:** ASD Participant Characteristics.

Variable	No Regression	Regression with Trigger	ASD without Trigger
Number of Cases	25	25	33
White, N (%)	22 (88%)	22 (88%)	31 (94%)
Males, N (%)	16 (64%)	21 (84%)	29 (88%)
Rural-urban commuting area codes	1.6 (2.1)	1.7 (1.8)	2.0 (1.8)
% Rural (≥6)	8%	8%	6%
Age at Regression		1y 4m (0y 9m)	1y 9m (1y 1m)

**Table 3:** Logistic Regression Model for Neurodevelopmental Regression without Trigger.

Model Term	β	Std. Error	t-Value	*p*	Odds Ratio (95% CI)
Intercept	−0.281	0.6539	−0.430	0.667	0.755 (0.210, 2.719)
**Season (Fall Reference)**					
Winter	−0.317	0.0336	−9.435	<0.0001	0.728 (0.682, 0.778)
Spring	0.090	0.0293	3.085	0.002	1.094 (1.033, 1.159)
Summer	0.220	0.0290	7.582	<0.001	1.246 (1.177, 1.319)
**NDR Event (Compare to Reference Time Period)**					
Weeks 1 to 2	0.248	0.2913	0.851	0.395	1.281 (0.724, 2.268)
Weeks 3 to 4	0.369	0.2865	1.288	0.198	1.446 (0.825, 2.535)
Weeks 5 to 6	0.693	0.3400	2.037	0.042	1.999 (1.027, 3.892)
**Air Pollution (PM2.5)**					
Overall Exposure	0.040	0.0018	21.847	<0.0001	1.041 (1.037, 1.044)
NDR Weeks 1 to 2	0.045	0.0163	2.750	0.006	1.046 (1.013, 1.080)
NDR Weeks 3 to 4	0.033	0.0149	2.237	0.025	1.034 (1.004, 1.064)
NDR Weeks 5 to 6	0.059	0.0165	3.591	<0.001	1.061 (1.027, 1.096)
**Ozone**					
Overall Exposure	0.010	0.0061	1.715	0.086	0.987 (0.985, 0.989)
NDR Weeks 1 to 2	0.009	0.0062	1.522	0.128	1.011 (0.999, 1.023)
NDR Weeks 3 to 4	0.023	0.0070	3.234	0.001	1.009 (0.997, 1.022)
NDR Weeks 5 to 6	0.010	0.0061	1.715	0.086	1.023 (1.009, 1.037)
**Maximum Temperature**					
Overall Exposure	−0.008	0.0008	−10.346	<0.0001	0.992 (0.990, 0.993)
NDR Weeks 1 to 2	−0.017	0.0044	−3.725	<0.001	0.984 (0.975, 0.992)
NDR Weeks 3 to 4	−0.016	0.0048	−3.437	<0.001	0.984 (0.975, 0.993)
NDR Weeks 5 to 6	−0.032	0.0055	−5.785	<0.001	0.969 (0.958, 0.979)

**Table 4:** Logistic Regression Model for Neurodevelopmental Regression with Trigger.

Model Term	Β	Std. Error	t-Value	*p*	Odds Ratio (95% CI)
Intercept	−2.304	0.7648	−3.012	0.003	0.100 (0.022, 0.447)
**Season (Fall Reference)**					
Winter	−0.124	0.0383	−3.248	0.001	0.883 (0.819, 0.952)
Spring	0.235	0.0337	6.958	<0.001	1.265 (1.184,1.351)
Summer	0.176	0.0339	5.193	<0.001	1.192 (1.116, 1.274)
**Maximum Temperature**					
Overall Exposure	0.012	0.0009	13.518	0.000	1.012 (1.010, 1.014)
**Ozone**					
Overall Exposure	−0.029	0.0009	−34.610	0.000	0.971 (0.969, 0.973)
**Precipitation**					
Overall Exposure	−0.315	0.0308	−10.253	0.000	0.729 (0.687, 0.775)

## Data Availability

Data is available upon request.
